# Resveratrol Nanocrystal Incorporated into Mesoporous Material: Rational Design and Screening through Quality-by-Design Approach

**DOI:** 10.3390/nano12020214

**Published:** 2022-01-10

**Authors:** Ahmad Ainurofiq, Yuniawan Hidayat, Eva Y. P. Lestari, Mayasri M. W. Kumalasari, Syaiful Choiri

**Affiliations:** 1 Pharmaceutical Technology and Drug Delivery, Department of Pharmacy, Universitas Sebelas Maret, Ir. Sutami 36A, Surakarta 57126, Indonesia; rofiq@mipa.uns.ac.id; 2 Department of Chemistry, Universitas Sebelas Maret, Ir. Sutami 36A, Surakarta 57126, Indonesia; yuniawan.hidayat@staff.uns.ac.id; 3 Department of Pharmacy, Universitas Sebelas Maret, Ir. Sutami 36A, Surakarta 57126, Indonesia; evayuliaputri@student.uns.ac.id (E.Y.P.L.); mayangmangesti08@student.uns.ac.id (M.M.W.K.)

**Keywords:** nanocrystal, resveratrol, fractional factorial design, polymers

## Abstract

Bioflavonoids from grape seeds feature powerful antioxidant and immunostimulant activities, but they present problems related to solubility and bioavailability. Nanocrystal (NC) incorporated into a mesoporous carrier is a promising strategy to address these issues. However, the preparation of this formulation involves the selection of factors affecting its critical quality attributes. Hence, this study aimed to develop an NC formulation incorporating resveratrol into a soluble mesoporous carrier based on rational screening design using a systematic and continuous development process, the quality-by-design paradigm. A mesoporous soluble carrier was prepared by spray-drying mannitol and ammonium carbonate. The NC was obtained by introducing the evaporated solvent containing a drug/polymer/surfactant and mesoporous carrier to the medium. A 2^6−2^ fractional factorial design (FFD) approach was carried out in the screening process to understand the main effect factors. The type and concentration of polymer and surfactant, resveratrol loading, and solvent were determined on the NC characteristics. The results indicated that drug loading, particle size, and solubility were mainly affected by RSV loading, PEG concentration, and Kolliphor EL concentration. The polymer contributed dominantly to reducing the particle size and enhancing solubility in this screening design. The presence of surfactants in this system made it possible to prolong the supersaturation process. According to the 2^6−2^ FFD, the factors selected to be further developed using a statistical technique according to the quality-by design-approach, Box Behnken Design, were Kolliphor EL, PEG400, and RSV loading.

## 1. Introduction

Antioxidant and immunostimulant activities have been the subject of increasing interest in recent years. These activities have received significant consideration, particularly for the preventive and curative treatment of the Coronavirus disease, 2019 (COVID-19) [1,2]. Furthermore, other studies of these activities have also increased dramatically during the last decade. Bioflavonoids were reported that were proven to be antioxidant and immunostimulant agents [3,4,5]. In addition, the improvement of several conditions, such as hypertension, hyperlipidemia, diabetes mellitus, cancer, cardiovascular diseases, neurodegeneration, aging, and antiviral and antibacterial properties, is affected by these activities. Therefore, further development of bioflavonoids is a promising candidate for the prevention and the improvement of the condition of COVID-19 patients [5,6]. The search for a new and potent bioflavonoid is intensively conducted, but it is inefficient due to time and costs [7]. Hence, drug development and discovery, as well as paradigm-shifting through drug delivery modification, can be proposed to enhance identified bioflavonoids. The major issue of bioflavonoids is the solubility and efflux substrate. The low solubility of bioflavonoid compounds has received consideration due to the resultant low bioavailability and its decrease of potential activity [8]. On the other hand, due to the contribution of a hydroxyl group in the bioflavonoid structure, it is susceptible to efflux transporter [9]. Therefore, enhancing solubility is the main action for enhancing bioflavonoid compounds’ antioxidant and immunostimulant activity.

Resveratrol (RSV), a bioflavonoid from grape seed, has been reported to offer powerful antioxidant and immunostimulant activity [10]. A comprehensive review regarding the number of publications containing RSV was reported. It offers numerous potential health benefits due to its antioxidant activity [11]. However, this molecule has received particular consideration due to its limited solubility. Therefore, as mentioned above, the bioavailability of this compound should be enhanced. Several strategies have been reported, including nanoemulsion [12], nanosuspension [13], and solid lipid formulations [14]. However, these technologies lack stability and are generally voluminous. In addition, the most feasible technology for scale-up and manufacturability is faced with a significant challenge. Moreover, conventional nanocrystal (NCs) in suspension (nanosuspension) faces a similar problem. The solidification of suspension-based NCs promotes aggregation and lacks nano-sized re-dispersibility [15].

Further development can be conducted by altering the solid-based formulation without altering the nano-sized features. This approach can be applied by a high surface-modified carrier, namely a mesoporous carrier. In addition, the use of inorganic material, such as silica or titanium, as a mesoporous carrier is extensively reported [16,17]. However, it features numerous problems; for example, its insoluble characteristic promotes alterations in the nano-redispersion capability and nanotoxicity issues due to bio-deposition and biocompatibility [18]. Therefore, a modified soluble mesoporous carrier is the best choice for solid nanocrystals incorporated into the carrier system.

The incorporation of drug stabilizers along with surfactants and polymers into the mesoporous carrier can be implemented [19]. Dissolved drugs in appropriate solvents stabilized by polymers and surfactants will fill the mesoporous pore. The evaporation of the solvent promotes the formation of NCs [20]. It features low thermodynamic stability due to its high Gibbs free energy; thus, it requires stabilization. Surfactants and polymers play fundamental roles in this system by preventing crystal growth and stabilizing the system when the solid carrier is introduced into the medium [21,22]. Anionic polymers and surfactants mainly act as steric hindrances and stabilize the nano-dispersion through this mechanism [23]. The most significant hurdle is how to select appropriate surfactants and polymers due to the large number of alternatives without any deterministic guidance to support the decision. On the other hand, the trial-and-error approach is a time-consuming and non-simultaneous assessment. Not only the surfactants and polymers, but also the concentration and amount of drug loading significantly impact the quality target profile of NCs using the evaporation technique [21]. Therefore, a technique to select and assess NC preparation factors that offer an efficient and effective factor screening and selection process is required.

Fractional factorial design (FFD), a quality-by-design approach, has been successfully applied for screening and factor in pharmaceutical preparations [24,25]. It offers rational design and scientific proof of the effective and efficient screening technique based on categorical and numerical factors. Thus, the novelty of this study is its development process of NCs incorporated into a mesoporous carrier through a simultaneous assessment of the screening process. In addition, an understanding and elucidation of the effect of the formulation factor on the critical quality attributes of NC preparation are presented. Therefore, this study aims to develop an NC formulation containing RSV incorporated into a soluble mesoporous carrier, based on a rational screening design using a systematic and continuous development process, the quality-by-design paradigm.

## 2. Materials and Methods

### 2.1. Material

RSV from grape seed (98%) was purchased from Thanen Chem (Changzhou, China). Kolliphor EL, Kolliphor P188, Kolliphor P407, Kolliphor RH40, and Kollidon K30 were obtained from BASF (Ludwigshafen, Germany). Propylene glycol, polyethylene glycol (PEG) 400, acetone, methanol, dichloromethane, ethanol, and ammonium carbonate were obtained from Merck (Darmstadt, Germany). Tween 80 and HPMC K100LV were obtained from Sigma Aldrich (St. Louis, MO, USA) and Colorcon (West Point, PA, USA), respectively. Mannitol was obtained from Roquette (Lestrem, France)

### 2.2. Preparation of Soluble Mesoporous Carrier

The mesoporous mannitol (MM) carrier was prepared using the spray drying technique. A solution containing a total solid of 10% (mannitol 92.5% and ammonium carbonate 7.5%) was used as a sample in the spray drying process using a Buchi mini-spray dryer B-290 (Flawil, Switzerland). The condition was adjusted at an inlet temperature of 120 °C, air pressure of 1.5 Bar, and a 2 mm nozzle, and the outlet temperature depended on the inlet temperature and airflow. The feeding rate was maintained at 5 mL/min. The sample was obtained from the collecting chamber and stored in a desiccator for further characterization and application. The mesoporous material was characterized by thermal behavior using differential scanning calorimeter and thermal gravimetric analysis, morphological evaluation using scanning electron microscopy, and vibrational spectroscopy.

### 2.3. Screening Design

A 2^6−2^ fractional factorial design was applied to screen appropriate solvents, polymers, and surfactants. A 16 run was constructed according to this design, and it is presented in Table S1 (Supplementary Materials). In addition, a graphical scheme of the study design is also presented in Figure S1. Prior to the design, miscibility and solubility among excipients were studied to determine the selected excipient used in the screening design. Kolliphor EL and Kolliphor P188 were used as surfactants, Kolliphor P407 and PEG 400 were utilized as polymers, and acetone and methanol were selected as solvents. Six factors, along with numerical and categorical factors, were used, i.e., polymer type and concentration, surfactant type and concentration, drug loading, and solvent type. Each factor featured two levels, i.e., high and low levels, which were coded as +1 and −1, respectively. The screening was conducted depending on the main effect of each factor on drug content, thermodynamic and kinetic solubilities, and particle size formation.

### 2.4. Preparation of Nanocrystal Incorporated into the Mesoporous Carrier

Amount of drug loading, type and concentration of both polymer and surfactant, and solvent were weighed according to Table S1. RSV was dissolved in solvent until a homogeneous solution was achieved. Surfactant and polymer were added gently until all components dissolved completely. A 20% (*w/v*) mesoporous carrier was added to the solution. The mixture was stirred for 12 h to ensure the solution full-filled the mesoporous of the carrier. Filtration was conducted to obtain a solid carrier containing RSV, and the residual solvent was eliminated by evaporation under heating up at 30 °C for one h. The sample was stored at ambient temperature and protected from light in the desiccator for further evaluation.

### 2.5. Resveratrol Assay in the Mesoporous Carrier

RSV contained in a gram of mesoporous carrier was determined spectrophotometrically. A 50.0 mg sample was weighed accurately and dissolved in methanol in an ultrasonic bath. Centrifugation was carried out to separate the supernatant and insoluble carrier (due to the limited solubility of mannitol in organic solvent). The supernatant was withdrawn, diluted appropriately, and analyzed at a wavelength of 315 nm. The amount of RSV in the solid mesoporous carrier was calculated according to the previously validated analytical methods along with an equation of *y* = 0.567*x* + 0.002 (coefficient determination, R^2^; accuracy; and precision of 0.998; 99.23%; and 1,69%, respectively).

### 2.6. Solubility Evaluation

Solubility evaluation was carried out according to the kinetic and thermodynamic solubility parameters. An excess amount of NC incorporated into MM (300 mg) was added to 5 mL of water. The sample was stirred for 48 h. A 0.2 mL sample was withdrawn at 1, 3, 5, 7, 10, 15, 20, 30, 45, and 60 min to evaluate the kinetic solubility. The saturated solubility was carried out after stirring for 48 h. The solubility characterization was evaluated at ambient conditions (25 ± 1 °C and RH 60 ± 10%). The sample was analyzed using spectrophotometrics at 305 nm, along with a validated analytical method.

### 2.7. Particle Size Evaluation

A total of 50 mg of NC incorporated into MM was weighed accurately. Thereafter, it was introduced into water at a stirring rate of 100 rpm for 30 min. The particle size of nano-dispersed was evaluated using a Malvern Particle Size Analyzer (Malvern, UK). The measurement was performed using a dynamic light scattering technique based on photon correlation spectroscopy at a wavelength, angle, and refractive index of 632 nm, 173° and 1.333, respectively. The sample absorbance was adjusted according to the absorbance of the sample. Gate time was adjusted in a range of 2.56 to 10.24 µs.

### 2.8. Nanocrystal Evaluation

RSV NC incorporated into mesoporous mannitol carrier was characterized. Thermal analysis, morphological analysis, and vibrational spectroscopy were applied. A simultaneous thermal analyzer LINSEIN ST1600 (Robbinsville, NJ, USA) comprising differential scanning calorimetry (DSC) and thermal gravimetric analysis (TGA) was applied in this study. An approximately 15–20.0 mg sample was placed into an aluminum pan and heated from ambient temperature (25 °C) to 400 °C at a ten °C/min rate. An empty pan was used as a reference.

Morphological analysis was carried out by Jeol JCM-7000 scanning electron microscopy (Tokyo, Japan). Samples were coated with gold for 100 s. Samples were observed at an accelerating voltage of 15 kV along with several magnifications until the desired picture was obtained. An Agilent Cary 630, attenuated total reflectance Fourier transform infrared (ATR-FTIR) spectrophotometer (Santa Clara, CA, USA) was used to characterize the vibrational interaction between the carrier, stabilizer, and NC. A sample was placed on the ATR crystal and scanned from 650–4000 cm^−1^ along with resolution and iteration of 2 cm^−1^ and 32 times, respectively.

### 2.9. Statistical Analysis

The obtained data were summarized and categorized according to response. The data of each response was fitted to Equation (1).

Y = β + a × A + b × B + c × C + d × D + e × E + f × F(1)

The models, including the intercept (β) and main effect coefficient (a, b, c, d, e, and f), were generated for all response variables by using multiple linear regression analysis (MLRA). A, B, C, D, E, and F were the levels of each factor. The models were evaluated based on several statistical parameters, including coefficient of determination (R^2^), adjusted coefficient of determination (Adj. R^2^), predicted coefficient of determination (Pred. R^2^), adequate precision (AP), and predicted residual error sum of squares (PRESS). All parameters were calculated depending on the data set in the screening design using Design Expert^®^ software version 11 (Stat-Ease Inc., Minneapolis, MN, USA). Factor contribution can be calculated using Equation (2) based on the percentage of each main effect (*m*) to the total coefficient of the main effects [25].

(2) $$\mathrm{Main}\;\mathrm{effect}\;\mathrm{contribution}\;(\%)=\frac{m_{x}}{\sum_{6}^{1}m}\;\times \;100\%$$

## 3. Result and Discussion

The intended purpose of this study was to provide a better understanding of the effect of factors affecting the quality target product profiles of the NC incorporated into mesoporous carriers through a simultaneous assessment of the screening design. In consequence, the study was initiated by the preparation of a mesoporous soluble carrier, prior screening of candidate factors, and screening model, as well as elucidating the factor effects, following the determination of selected factors.

### 3.1. Mesoporous Mannitol Preparation

MM, a soluble carrier, was applied to carry and stabilize the NC formulation. The MM ideally featured a spherical geometry, along with a hollow core and a porous wall structure. During the spray drying process, it was prepared by sublimating the pore promoting agent, ammonium carbonate (AC) [26,27]. The morphological analysis using SEM indicated that the aforementioned characteristics were achieved. The thermal analysis proved that the AC completely disappeared (Figure S2 in Supplementary Materials). The thermal gravimetric showed that the sublimation of AC proceeded through three stages and sublimated completely at 145.6 °C. Meanwhile, the mannitol featured a single melting point at 174.21 °C. The MM featured different peak characteristics. A broadening peak and an overlaid double peak were observed in MM (174.89 and 179 °C, respectively). This confirmed an alteration in the crystal lattice structure, the development of amorphous material due to the spray drying process.

### 3.2. Prior Screening Process by Factorial Design

The selection of studied factors is an important step during screening design. Screening design is intended to screen and select the most significant and valuable factor for further optimization during NC preparation. There were many polymer candidates, including Kolliphor P407, Kollidon K30, HPMC K100 LV, and PEG400. Meanwhile, Kolliphor P188, Tween 80, Kolliphor EL, Labrafil M1944 CS, and Kolliphor RH40 were surfactants, and propyleneglycol was applied as a co-surfactant. In addition, the preparation process required volatile solvents, i.e., methanol, ethanol, dichloromethane, and acetone. All the components, polymers, surfactants, and solvents, must offer good solubility and miscibility. Therefore, a preliminary FFD step should be conducted to select appropriate screening factors in a NC system [25]. The solubility of polymers and surfactants in several organic solvents was the primary concern in this study. Therefore, this design selected Kolliphor P407, Kolliphor P188, PEG 400, and Kolliphor EL as polymers and surfactants due to the excellent solubility and miscibility between the solvent and the RSV.

### 3.3. Preparation and Characterization of Resveratrol Nanocrystal Incorporated into MM

The preparation process was based on incorporating the solution into the mesoporous structure of MM. Volatile solvents were eliminated by ambient drying [27]. NCs formed due to the evaporation of the solvent, and nucleation began. Furthermore, the NC was stabilized by the surfactant and polymer in the system [21]. In order to ensure NC formation and the assessment of the interaction between the NC and the carriers, several characterizations were performed.

**Figure 1:**
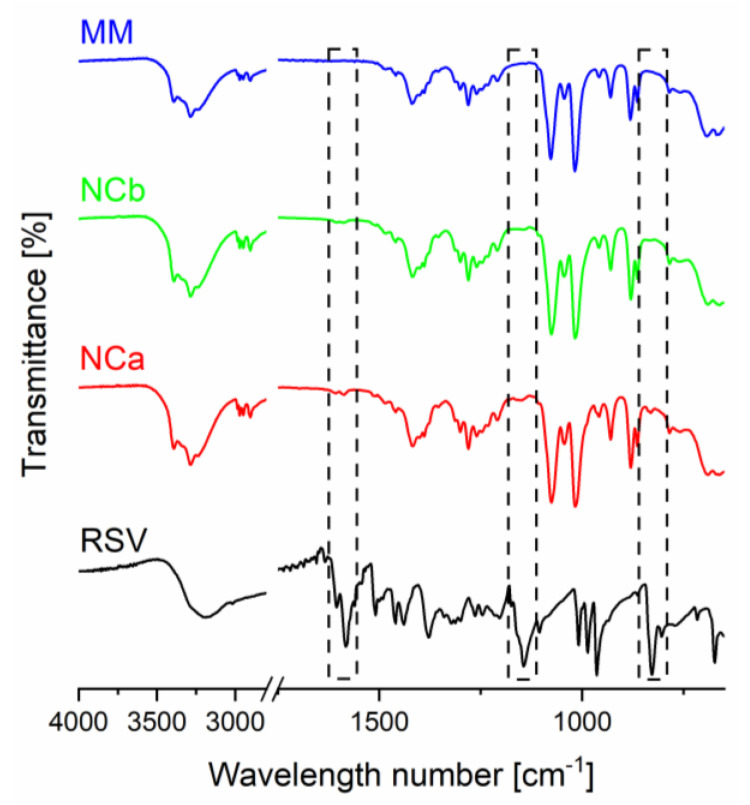
ATR-FTIR spectra of resveratrol (RSV), mesoporous mannitol (MM), nanocrystal incorporated into mesoporous mannitol (NC; NCa, and NCb for Runs 15 and 12, respectively).

The ATR-FTIR spectra of RSV, MM, and NC incorporated into MM are presented in Figure 1. The vibrational spectra identified the molecular interaction between the NCs and the carrier. The MM spectra consisted of several specific vibrational peaks, i.e., hydroxyl, alkyl groups, and C-O vibrational peaks at 3884, 1416, and 1077 cm^−1^, respectively. Broadening the specific functional peaks confirmed that the MM took an amorphous form [28]. The FTIR spectra of the NCs displayed a similar pattern to the MM spectra. This phenomenon was affected by an additive spectra effect due to the proportion of RSV in the system [29]. The RSV loading was around 20–80 mg/g (2–8%, *w/w*). Therefore, it featured a small contribution of RSV in its spectra. Hence, the spectra of the NCs were almost similar to the MM. RSV demonstrated specific vibrational peaks, i.e., a carbonyl vibrational peak at 1604 cm^−1^, a C-O vibrational peak at 1144 cm^−1^, and an alkyl bending vibrational peak at 829 cm^−1^. These native peaks were slightly retained in the NCs’ spectra. Moreover, the broadening and shifting of peaks from the native MM spectra were also not observed. These results confirmed that there was no interaction between the carrier and the RSV. In addition, there was no addition of a new peak, confirming the degradation of the RSV. However, this study was limited by the interaction between the carrier and the NCs. The interactions between the polymers/surfactants and the drugs in the system should be considered. However, they could not be assessed simultaneously in this study due to the preparation process of the NCs incorporated into the MM carrier.

**Figure 2:**
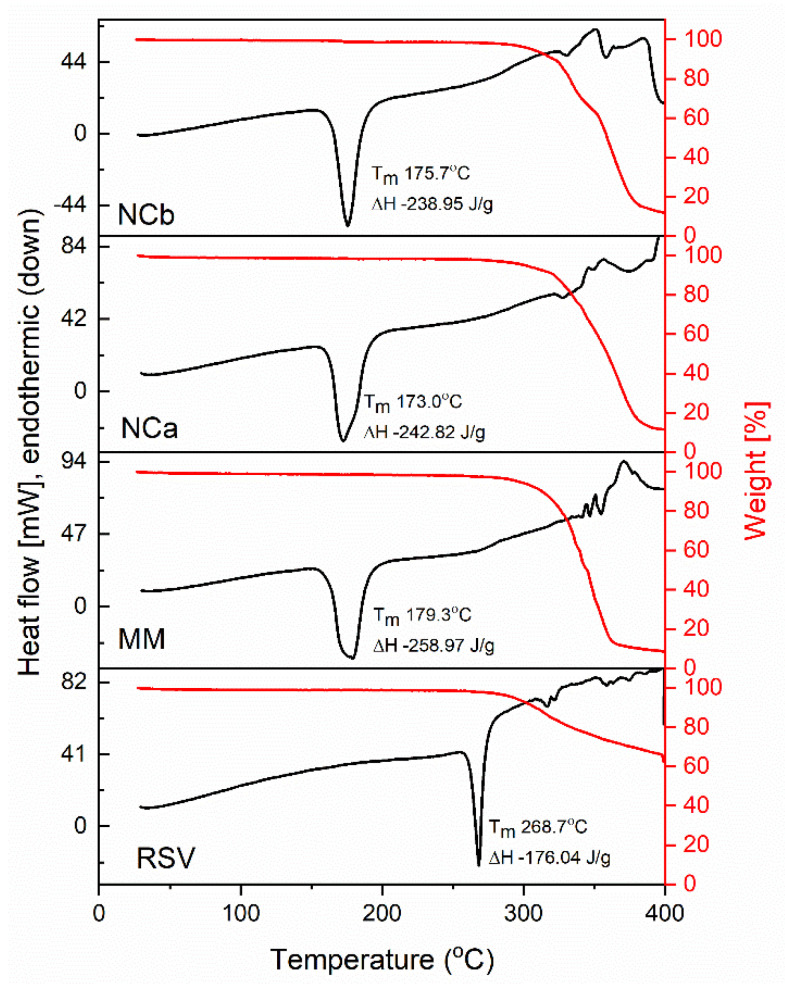
Thermogram of DSC (black line) and TGA (red line) of resveratrol (RSV), mannitol mesoporous (MM), and nanocrystal (NC, NCa, and NCb for Runs 12 and 15, respectively).

The thermal profiles of the RSV, MM, and NCs are depicted in Figure 2. The RSV featured a sharp endothermic peak at 268.4 °C, corresponding to the melting phenomenon, followed by decomposition. It lost about 40% of its mass during heating at 400 °C. The thermal MM featured a relatively broad endothermic peak at 179 °C and an overlaid peak at 174.9 °C. It was affected by the mannitol’s amorphous form during the spray drying process. In addition, it underwent a decomposition of 88% at 365.4 °C. A narrower peak than that of the MM was observed in the NC thermogram. However, different drug loadings exerted different effects on the thermogram. Higher drug loadings created sharper endothermic peaks. The NCa melted at 173 °C. Meanwhile, the NCb displayed an endothermic peak at 165.7 °C. There was an interaction between the system and the carrier, i.e., the thermal stability changed along with a decomposition of 85% around 390 °C. In addition, different drug loadings shifted the melting point of the carrier. These interactions were limited by the physical interaction of the attachment of the nanoparticle crystal in the surface of the MM. Therefore, the interaction was not observed in the FTIR results (molecular interaction).

**Figure 3:**
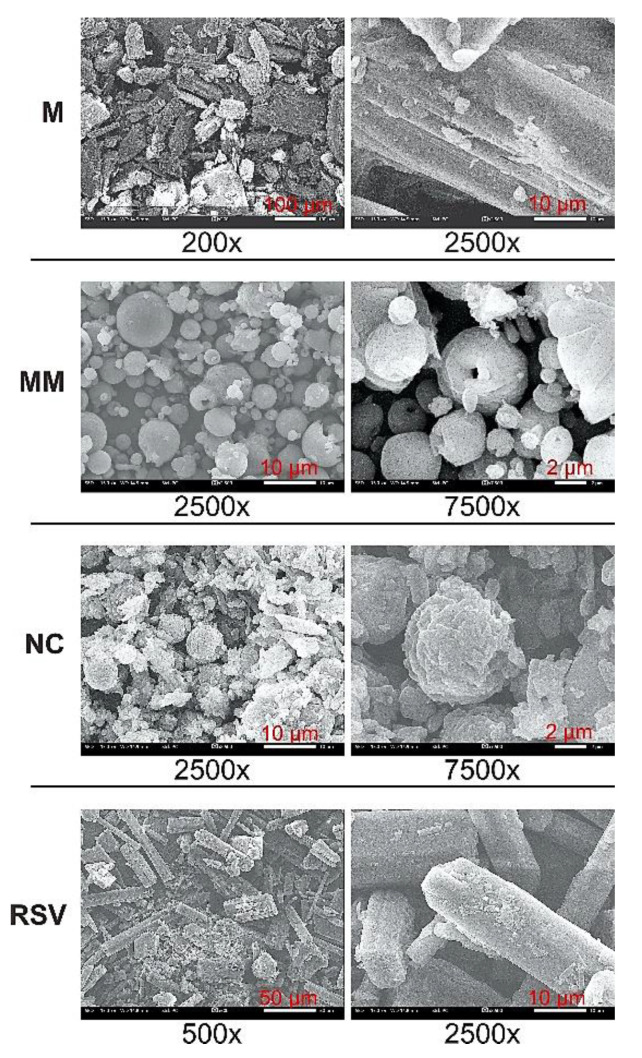
SEM photograph of resveratrol (RSV), mannitol (M), mesoporous mannitol (MM), and nanocrystal (NC, Run 15).

The morphological photographs of the RSV, mannitol, MM, and NC are depicted in Figure 3. The morphological of photograph of the RSV shows that it featured a rod-like structure with a smooth surface. Meanwhile, the mannitol displayed an irregular rod-like structure along with a slab structure. The MM was completely spherical (1–10 µm), and featured a porous surface and a hollow inner core. The incorporation of MM in solution slightly altered the structure of MM, but generally, it maintained its native structure. Moreover, modifications on the surface were observed due to the NCs in the RSV. The solubilized RSV was distributed in both the surface and the inner core of the MM, as well as the mesoporous system. Thereafter, the solvent was evaporated, and the formation of NCs depended on the mesoporous structure stabilized by the polymer and surfactant. Therefore, the RSV NCs were placed into the porous areas and on the surface of the outer and inner mannitol wall due to the spherical and hollow structure of the MM. This result was confirmed by the slight alteration in the surface of the NC, i.e., a slightly rough surface was observed due to the attachment of the NCs.

### 3.4. The Effect on Drug Loading

Drug loading is a primary requirement for drug delivery. The higher the drug loading, the more efficient the system [20]. In the NC, through the solvent evaporation technique, the drug loading depended on the porous system, viscosity, and degree of volatility of the solvent. The drug loadings of all the runs were in the range of 15.2–100.2 mg/g. The wide range of the drug loading in the system was significantly affected by the studied factors. According to the MLRA of the drug loading parameters (Table 1), it could be inferred that the studied factor significantly affected the drug loading by 95.29% (*p* < 0.05). The model was good at prediction due to the small gap between adj. R^2^ and Pred. R^2^. This was where the concentration of RSV (*p* < 0.05) and solvent (*p* < 0.1) significantly affected the drug loading.

**Figure 4:**
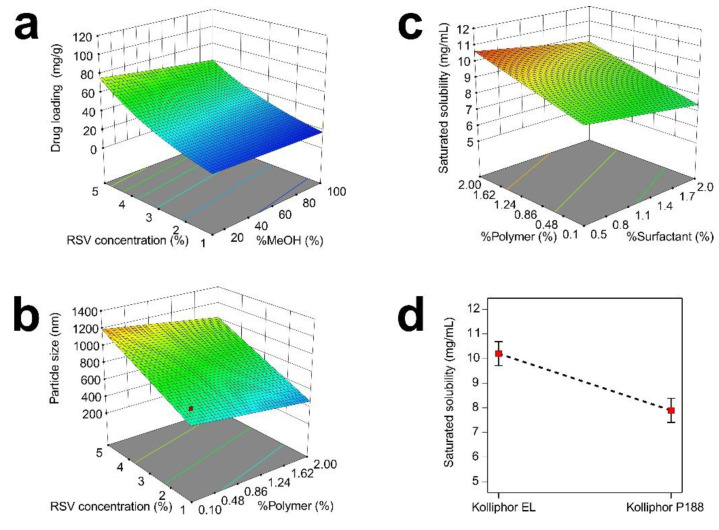
Contour plot of drug loading (**a**), particle size (**b**), and saturated solubility (**c**); and single plot of type of surfactant (**d**).

However, the contribution of the concentration of RSV was almost seven times higher than that of the solvent. Greater RSV concentrations in the system promoted higher drug loading into the porous system. Moreover, increasing the methanol concentration in the solvent reduced the drug loading. It was affected by the solubility of the RSV in the mixture [30]. The amount of drug incorporated into the mesoporous system depends on the solvent-containing formulation’s ability to penetrate into the porous system deeply. The surface tension is not a significant problem due to the containment of the solvent and surfactants/polymers. Therefore, only the drug concentration affected by the amount of drug added and solubility in appropriate solvent governs the drug loading in the NCs incorporated into the mesoporous carrier system [31]. In order to investigate the effect of RSV concentration and methanol concentration in acetone, a contour plot of DL was constructed (Figure 4a). It could be concluded that the highest drug loading was obtained at the highest concentration of RSV and the lowest methanol concentration in acetone. The lowest RSV concentration featured a similar value of drug loading under different methanol concentrations. In terms of types and concentrations, the polymers and surfactants were not considered in the screening design due to their insignificant effects on the drug loading.

### 3.5. The Effect on Particle Size

NC was produced when the solid system was introduced into the water. Due to the soluble carrier, the entrapped NC in the porous system formed the nano-dispersion stabilized by the surfactants and polymers. The particle size depended on the NC formed during the evaporation of the liquid system and the dilution process [15]. Generally, a particle size of less than 1000 nm is acceptable for bioavailability enhancement [23]. The particle sizes of all the runs varied from 309.9 to 1371.3 nm. In order to understand the effect of the screened factors, the MLRA approach was applied (Table 1). The studied factors affected the particle size significantly (*p* < 0.05), by 89.96%. According to the model, only the RSV and polymer concentrations significantly affected the particle size (*p* < 0.05). However, the methanol concentration was slightly significant (*p* < 0.1). The factor contributions showed that the effect of RSV loading was three times higher than that of the polymer and methanol concentrations. In addition, the RSV concentration increased the particle size. Therefore, the greater the RSV concentration, the larger the particle size. The higher drug loading promoted a greater supersaturation degree during solvent evaporation, and thus the tendency towards crystal growth was induced [32]. This phenomenon was confirmed by the main effects’ coefficient of drug loading.

On the other hand, increasing the polymers and methanol concentrations reduced the particle size. Higher concentrations of molecular dispersed drugs promoted a greater tendency towards nucleation. Meanwhile, the use of polymers as stabilizers reduces the particle size by inhibiting the nucleation process through steric hindrance [22,23]. In addition, the surfactant that provided a shielding effect to avoid particle enlargement exerted no significant effect (*p* > 0.05). Moreover, different polymer and surfactant types exerted no significant effect on particle size (*p* > 0.05). Figure 4b shows the simultaneous assessment of the RSV and polymer concentrations on particle size. The smallest particle size was obtained at the lowest RSV loading and the highest polymer concentration. The slope of the factor changes shows that the RSV loading mainly affected the enlargement of the particle size. The screening model was limited to studying the interaction between factors. Therefore, it should be considered for further development.

### 3.6. The Effect on the Solubility

The solubility was observed regarding the thermodynamic and kinetic solubilities. The thermodynamic solubility, saturated solubility, was applied to assess the effect of solubility enhancement [33,34]. Generally, NC produced a dramatic enhancement of solubility [35,36]. On the other hand, the kinetic solubility reflected the supersaturation phenomenon and its stabilization. Kinetic solubility is a primary attribute of bioavailability enhancement due to the greater absorption of drugs when supersaturation is reached [37]. Meanwhile, the thermodynamic form of solubility, saturated solubility, is more straightforward to observe than kinetic solubility. The saturated solubility of the RSV varied from 5.25 to 11.04 mg/mL. Meanwhile, the saturated solubility of the plain RSV was 32.08 ± 2.53 µg/mL. Therefore, the NC formulation enhanced the solubility of RST by 163-to-344 fold. The data were fitted to the MLRA equation (Table 1) and the model was significant (*p* < 0.05), along with the factor affecting the saturated solubility by 87.42%. In addition, the model was adequate due to the small gap between adj. R^2^ and pred. R^2^, of less than 0.2. According to the equation and contribution, the type and concentration of surfactant, as well as the concentration of polymer, significantly affected the saturated solubility (*p* < 0.05). The surfactant type dominantly affected the saturated solubility by 34.68%. The saturated solubility using Kolliphor EL, along with all the different combinations of other factors, was higher than that of Kolliphor P188 (Figure 4c). It was affected by the native structure of the surfactant. Kolliphor EL was the native surfactant derived from Castrol oil. Meanwhile, Kolliphor P188 is an amphiphilic polymer applied as a surfactant. However, the different polymer types did not affect the saturated solubility.

The concentrations of the polymers and surfactants also affected the saturated solubility. The contribution of the polymer concentration was twice that of the surfactant concentration. However, the polymer concentration exerted a positive effect on enhancing the saturated solubility. Meanwhile, the surfactant concentration reduced the saturated solubility. The highest saturated solubility was obtained at the highest and lowest polymer and surfactant concentrations, respectively (Figure 4d). The effect of surfactant concentration was higher at high levels of polymer concentration. Therefore, Kolliphor EL and its concentration and the polymer concentration were considered to enhance saturated solubility.

**Figure 5:**
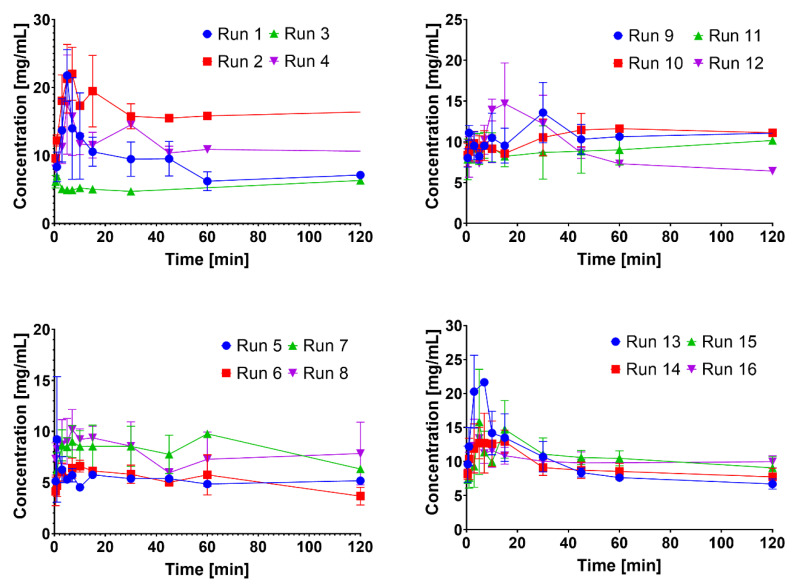
The kinetic solubility of nanocrystal incorporated into mesoporous material using fractional factorial design.

The kinetic solubility was also studied in this screening study. The kinetic solubility (not sink condition) was more meaningful than *in vitro* dissolution for assessing the effect of particle size on the NC quality target profiles [23]. The kinetic solubility profiles of all the runs is presented in Figure 5. The profiles showed that solubility enhancement was observed around 0–40 min, followed by a reduction in solubility. The solubility was affected by the precipitation phenomenon. Different runs (polymer and surfactant combination) resulted in different patterns. Runs 3–8 featured a low supersaturation degree due to their low concentrations of polymers (0.1%). The polymers and surfactants in this system are responsible for maintaining the supersaturation concentration through the steric hindrance and shielding effects, respectively [37,38]. Another low supersaturation effect was observed at Runs 9, 10, 11, and 15. It was due to either high drug loading or low methanol content in the solvent. Both factors enhanced supersaturation during the NC preparation. The solubility of the RSV in methanol was higher than that of the acetone [30]. On the other hand, high supersaturation profiles were observed at Runs 1, 2, and 12 due to the low level of RSV loading. However, the individual effect of each factor was challenging to interpret due to the design of the experiment. Moreover, the effect of the studied factor was easily identified by the equation of the model, particularly at supersaturation parameters, e.g., the solubility rate and degree during the supersaturation phenomenon.

**Figure 6:**
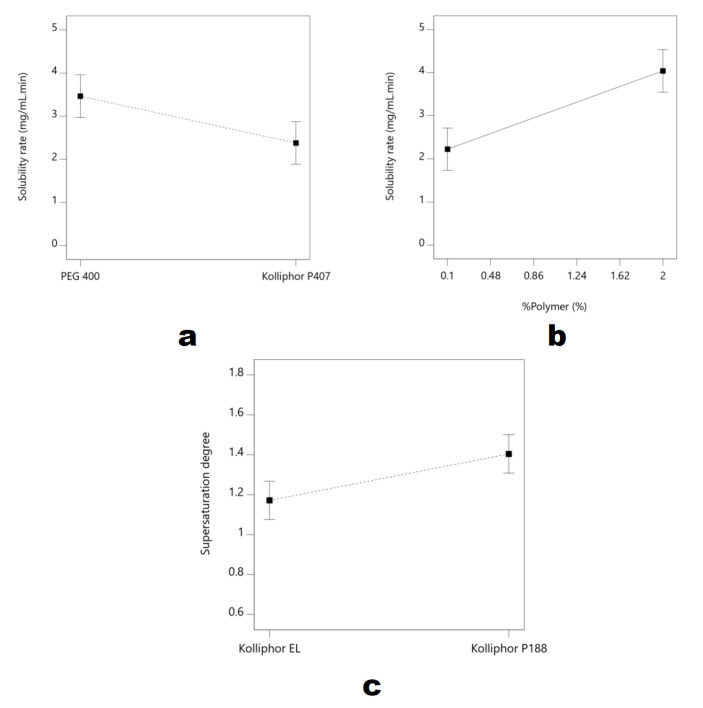
Single plots of solubility rate at polymer type (**a**) and concentration (**b**), and supersaturation degree at surfactant type (**c**).

The solubility rate of all the runs was in the range of 0.228 to 4.860 mg/mL/min. At the same time, the degree of supersaturation varied from 0.80 to 1.72. These models were significant (*p* < 0.05), along with the factor effect of 75.35 and 71.80% for solubility rate and supersaturation degree, respectively. Both models featured a large gap between adj. R^2^ and pred. R^2^. Therefore, it was particularly important to use these models carefully due to misleading predictions. According to the factor contribution, the factor contributing to the solubility rate differed from the saturation degree. The polymer type and concentration were the most significant factors affecting the solubility rate (*p* < 0.05). Meanwhile, the supersaturation degree was significantly affected by the type of surfactant and RSV loading (*p* < 0.05). The identification of dominant factors can be conducted using a single plot [24,25]. The polymer concentrations and types were considered to be the solubility rate parameters. Meanwhile, the surfactant type was the most dominant factor in the supersaturation degree. The PEG 400 featured a higher solubility rate compared to the Kolliphor P407 (Figure 6a). The higher the PEG 400 concentration, the faster the solubility rate (Figure 6b). According to the single plots, PEG was selected as the polymer based on the solubility rate parameter.

Kolliphor EL played a fundamental role in the stabilization of the solubilized RSV and reducing the precipitation phenomenon. Surfactants adsorb on the particle surface and build up the shield; this is known as the surfactant shielding effect. This effect prevents particle enlargement by reducing the Gibbs free energy. Smaller nano-sized particles feature greater amounts of Gibbs free energy [15,23,36]. This is unstable thermodynamically and produces a stronger saturation effect. However, the surfactant reduces the precipitation by stabilizing the dispersed nano-sized particle. This was proven by the supersaturation degree of the RSV (Figure 6c). The lower the supersaturation degree, the closer the gap between the concentration maximum at supersaturation and the saturated solubility. Therefore, Kolliphor EL was considered in maintaining the supersaturation step. In addition, the higher RSV concentration was more desirable due to the lower degree of supersaturation. Overall, the supersaturation degrees of all the runs were categorized as featuring a small gap, with not more than twice the supersaturated solubility.

### 3.7. The Effect on Zeta Potential

The zeta potential is a fundamental characteristic of nano-dispersion stabilization. The tremendous zeta potential provided better stability due to the repulsion force between particles. The zeta potential of all the runs was in the range of −33.7 to −11.1 mV. However, the zeta potential model in this screening design was not significant (*p* > 0.05). This revealed that zeta potential could not be applied as the screening parameter in this study. This phenomenon was affected by the adsorbed non-ionic surfactants and polymers on the particle’s surface. It exerted no charge effect on the particle dispersion. Hence, it exerted no significant effect on the alteration to the zeta potential. Only the RSV contributed to the zeta potential value due to the partial net negative charge in the structure.

### 3.8. Determining the Screening Design

According to the previously mentioned discussion on the several parameters in the screening design, the screening factor can be determined by selecting the significant factor affecting the critical quality attributes. Prior determination of the priority factor could help to determine the selected factor to be further developed. Particle size and solubility, both kinetic and thermodynamic, were the main concerns in developing the NC formulation. Therefore, these parameters received more significant consideration. The PEG concentration was the influential factor determining the particle size and solubility. The use of Kolliphor EL as a surfactant was the primary concern in maintaining the supersaturation degree. In addition, the RSV concentration contributed to the drug loading, particle size, and supersaturation degree. Depending on these results, it could be concluded that the selected factors in the screening design were the Kolliphor EL, PEG 400, and RSV concentrations.

## 4. Conclusions

A fractional factorial design was successfully applied for screening design in the preparation of NC incorporated into mesoporous mannitol containing RSV. This study thoroughly explained and elucidated the factors’ main effects on the NC preparation. However, the interactions between the factors could not be studied simultaneously due to the efficiency process in the screening design. Moreover, this technique could explain the effect of the selected factors on the screening design, particularly in the NC formulation. Practically, it could be applied in other pharmaceutical product screening developments.

The simultaneous assessment through drug loading, particle size, and solubility was mainly affected by RSV concentration, PEG concentration, and Kolliphor EL concentration. In this screening design, the polymers contributed dominantly to reducing the particle size and enhancing the solubility. The presence of surfactants in this system helped to prolong the supersaturation process. According to the fractional factorial design, the selected factors to be further developed using a comprehensive technique according to quality by design approach, Box Behnken Design, were Kolliphor EL, PEG400, and RSV concentration.

## Figures and Tables

**Table 1:** Statistical analysis, contribution, and goodness-of-fit parameters of screening design in nanocrystal incorporation into mesoporous mannitol using the fractional factorial design approach.

Parameters	Log (DL) × 10^−1^ (mg/g)		PS (nm)		SR [mg/mL. menit]		SS (mg/mL)		SD × 10^−1^		ZP (mV)	
	Coef.	Cont. (%)	Coef.	Cont. (%]	Coef.	Cont. (%)	Coef.	Cont. (%)	Coef.	Cont. (%)	Coef.	Cont. (%)
Intercept	+15.80	-	+794.31	-	+1.98	-	+8.32	-	+11.3	-	−21.65	-
A	−0.462 ^b^	−13.11	−70.75 ^b^	−14.73	−0.054	−2.55	+0.324	9.74	−0.19	−4.76	−1.54	−38.56
B	−0.004	−0.12	+41.41	8.62	−0.543 ^a^	−25.83	+0.086	2.60	−0.46	−11.43	−0.01	−0.16
C	+0.320	9.07	−6.61	−1.38	−0.383	−18.23	−1.15 ^a^	−34.68	+1.16 ^a^	28.67	+0.82	20.53
D	+0.020	0.56	−84.52 ^a^	−17.60	+0.908 ^a^	43.19	+1.09 ^a^	32.82	+0.25	6.17	+0.78	19.44
E	−0.005	−0.15	−0.9135	−0.19	−0.190	−9.06	−0.502 ^a^	−15.10	+0.49	12.03	+0.71	17.87
F	+2.714 ^a^	77.00	+275.99 ^a^	57.48	−0.024	−1.14	+0.168	5.06	−0.15 ^a^	−36.93	−0.14	−3.45
*p*-value model	<0.0001		0.0005		0.0209		0.0013		0.0356		0.9884	
R^2^	0.9529		0.8996		0.7535		0.8742		0.7180		0.0813	
Adj. R^2^	0.9229		0.8326		0.5891		0.7904		0.5300		−0.5311	
Pred. R^2^	0.8510		0.6826		0.2209		0.6025		1087		−1.9034	
AP	12.93		10.82		7.13		10.47		7.20		1.32	

DL = drug loading, PS = particle size, SR = solubility rate, SS = saturated solubility, SD = supersaturation degree, ZP = zeta potential, Coef. = coefficient of regression, Cont. = contribution of factor, A = % methanol, B = Polymers, C = Surfactant, D = % polymers, E = % surfactant, F = drug loading, ^a^ = *p* < 0.05, ^b^ = *p* < 0.1, R^2^ = coefficient of determination, Adj. R^2^ = adjusted R^2^, Pred. R^2^ = Prediction R^2^, and AP = adequate precision.

## Data Availability

Not applicable.

## Supplementary Materials

The following supporting information can be downloaded at: https://www.mdpi.com/article/10.3390/nano12020214/s1, Figure S1: Graphical scheme of study design; Figure S2: Thermogram of DSC (red-line) and TGA (black-line) of mannitol, ammonium carbonat (AC), and mannitol mesoporous (MM); Table S1: Design of experiment of 2^6-2^ fractional factorial design for screening studied factor on nanocrystal resveratrol incorporated into mesoporous mannitol.
