# Supplementary material for: Resveratrol Nanocrystal Incorporated into Mesoporous Material: Rational Design and Screening through Quality-by-Design Approach
Source: Nanomaterials (Basel). 2022 Jan 10;12(2):214. doi: 10.3390/nano12020214 (PMC8779882; doi:10.3390/nano12020214)
Supplement: Supplementary file 1 [file nanomaterials-12-00214-s001.zip › nanomaterials-1528667-supplementary.pdf]

nanomaterials

Supplementary material

Resveratrol Nanocrystal Incorporated into Mesoporous Material: Rational Design and Screening through Quality-by-Design Approach

Ahmad Ainurofiq [1], Yuniawan Hidayat [2], Eva Y. P. Lestari [3], Mayasri M. W. Kumalasari [3] and Syaiful Choiri [1,*]

[1] Pharmaceutical Technology and Drug Delivery, Department of Pharmacy, Universitas Sebelas Maret, Ir. Sutami 36A, Surakarta 57126, Indonesia; rofiq@mipa.uns.ac.id
[2] Department of Chemistry, Universitas Sebelas Maret, Ir. Sutami 36A, Surakarta 57126, Indonesia; yuniawan.hidayat@staff.uns.ac.id
[3] Department of Pharmacy, Universitas Sebelas Maret, Ir. Sutami 36A, Surakarta 57126, Indonesia; evayuliaputri@student.uns.ac.id (E.Y.P.L.); mayangmangesti08@student.uns.ac.id (M.M.W.K.)
* Correspondence: s.choiri@mipa.uns.ac.id

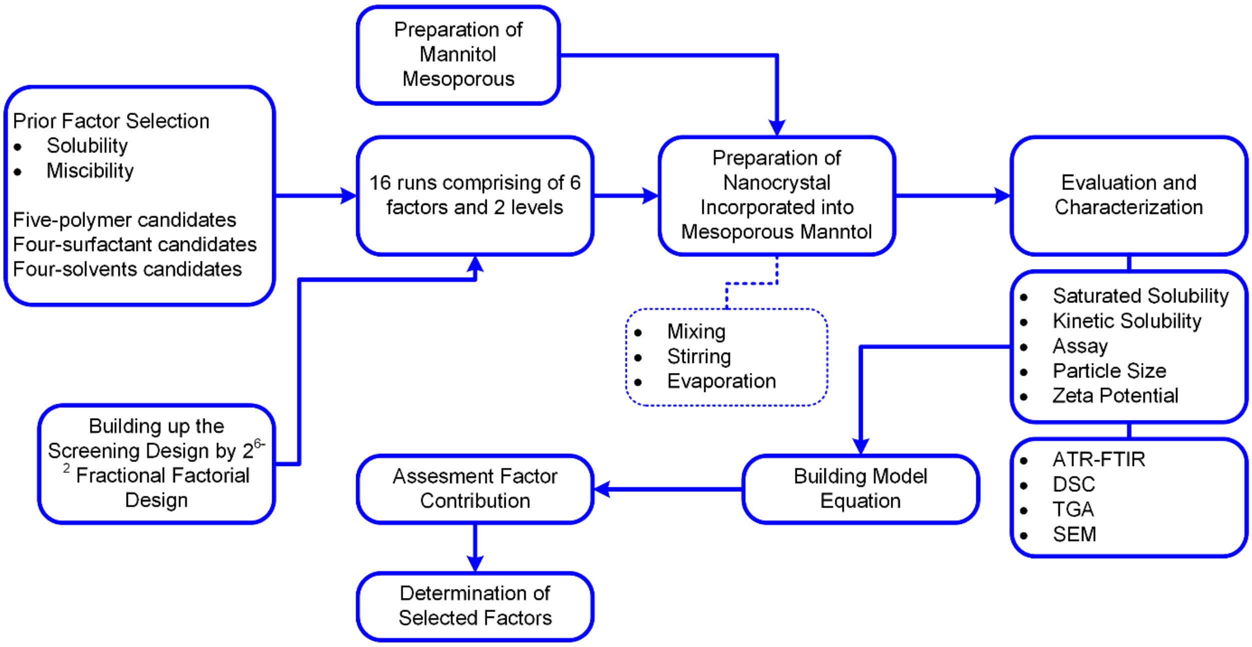

Figure S1. Graphical scheme of study design.

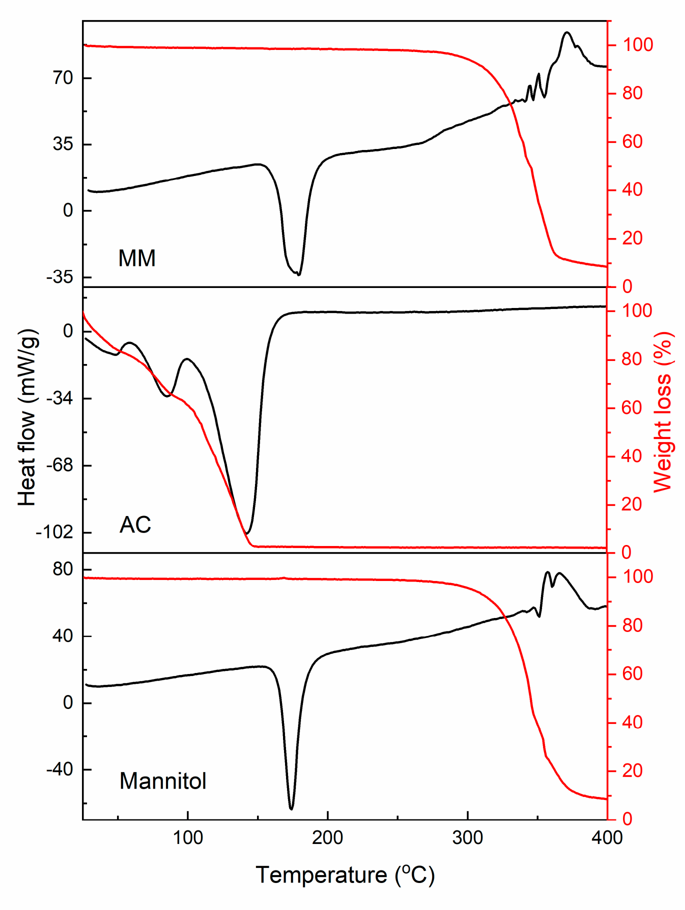

Figure S2. Thermogram of DSC (red-line) and TGA (black-line) of mannitol, ammonium carbonat (AC), and mannitol mesoporous (MM).

Table S1. Design of experiment of 2^{6-2} fractional factorial design for screening studied factor on nanocrystal resveratrol incorporated into mesoporous mannitol.

Runs	Coded						Actual					
	A [%]	B	C	D [%]	E [%]	F [%]	A [%]	B	C	D [%]	E [%]	F [%]
1	-1	{ -1 }	{ -1 }	-1	-1	-1	10	PEG 400	Kolliphor EL	0.1	0.5	1
2	1	{ -1 }	{ -1 }	-1	1	-1	100	PEG 400	Kolliphor EL	0.1	2	1
3	-1	{ 1 }	{ -1 }	-1	1	1	10	Kolliphor P407	Kolliphor EL	0.1	2	5
4	1	{ 1 }	{ -1 }	-1	-1	1	100	Kolliphor P407	Kolliphor EL	0.1	0.5	5
5	-1	{ -1 }	{ 1 }	-1	1	1	10	PEG 400	Kolliphor P188	0.1	2	5
6	1	{ -1 }	{ 1 }	-1	-1	1	100	PEG 400	Kolliphor P188	0.1	0.5	5
7	-1	{ 1 }	{ 1 }	-1	-1	-1	10	Kolliphor P407	Kolliphor P188	0.1	0.5	1
8	1	{ 1 }	{ 1 }	-1	1	-1	100	Kolliphor P407	Kolliphor P188	0.1	2	1
9	-1	{ -1 }	{ -1 }	1	-1	1	10	PEG 400	Kolliphor EL	2	0.5	5
10	1	{ -1 }	{ -1 }	1	1	1	100	PEG 400	Kolliphor EL	2	2	5
11	-1	{ 1 }	{ -1 }	1	1	-1	10	Kolliphor P407	Kolliphor EL	2	2	1
12	1	{ 1 }	{ -1 }	1	-1	-1	100	Kolliphor P407	Kolliphor EL	2	0.5	1
13	-1	{ -1 }	{ 1 }	1	1	-1	10	PEG 400	Kolliphor P188	2	2	1
14	1	{ -1 }	{ 1 }	1	-1	-1	100	PEG 400	Kolliphor P188	2	0.5	1
15	-1	{ 1 }	{ 1 }	1	-1	1	10	Kolliphor P407	Kolliphor P188	2	0.5	5
16	1	{ 1 }	{ 1 }	1	1	1	100	Kolliphor P407	Kolliphor P188	2	2	5

Note: A, Methanol concentration; B, type of polymers; C, type of surfactants; D, polymer concentration; E, surfactant concentration; and F, resveratrol loading. B and C are categorical factors. A, D, E, and F are numerical factors.
